# Dual-Functional Gel-Based Delivery of Chitosan-Coated Gold Nanoparticles for Accelerated Bone Healing in Defect Models

**DOI:** 10.3390/pharmaceutics18070843

**Published:** 2026-07-10

**Authors:** Noha M. Badawi, Shereen Nader Raafat, Mohamed M. Kataia, Caroline Maged Massieh, Sherihan Ahmed Sayed, Asmaa Saleh, Jawaher Abdullah Alamoudi, Hadeel A. Mousa

**Affiliations:** 1 Department of Pharmaceutics and Pharmaceutical Technology, Faculty of Pharmacy, The British University in Egypt, El-Sherouk City 11837, Cairo, Egypt; 2 Department of Pharmacology (Medical Sciences), Faculty of Dentistry, The British University in Egypt, El-Sherouk City 11837, Cairo, Egypt; shereen.nader@bue.edu.eg; 3 Dental Science Research Group, Health Research Centre of Excellence, The British University in Egypt, El-Sherouk City 11837, Cairo, Egypt; 4 Department of Endodontics, Faculty of Dentistry, The British University in Egypt, El-Sherouk City 11837, Cairo, Egypt; mohamed.kataia@bue.edu.eg; 5 Department of Oral Biology, Faculty of Dentistry, The British University in Egypt, El-Sherouk City 11837, Cairo, Egypt; caroline.maged@bue.edu.eg; 6 Department of Clinical Pharmacy and Pharmacy Practice, Faculty of Pharmacy, Galala University, New Galala City 43511, Suez, Egypt; sherihan.ahmed@gu.edu.eg; 7 Department of Pharmaceutical Sciences, College of Pharmacy, Princess Nourah bint Abdulrahman University, P.O. Box 84428, Riyadh 11671, Saudi Arabia; asali@pnu.edu.sa (A.S.); jaalamoudi@pnu.edu.sa (J.A.A.); 8 Department of Pharmaceutics (Medical Sciences), Faculty of Dentistry, The British University in Egypt, El-Sherouk City 11837, Cairo, Egypt; hadeel.mousa@bue.edu.eg

**Keywords:** gold nanoparticles, chitosan coating, localized delivery, osteogenic differentiation, radiographs, osteogenic markers

## Abstract

**Background**: Effective management of bone defects remains a major clinical challenge, driving continuous efforts to develop bioactive, localized delivery systems that support bone regeneration. Gold nanoparticles (AuNPs) have gained attention in regenerative medicine for their capacity to modulate cellular activity. Yet, their application in functional delivery systems for bone repair is still limited. Chitosan (CS), a naturally derived biopolymer, exhibits notable osteoinductive properties, particularly when used to modify nanoparticulate carriers. **Objectives**: In this study, AuNPs and chitosan-coated gold nanoparticles (CS-AuNPs) were formulated, characterized, and incorporated into gel preparations to evaluate their physicochemical properties and therapeutic potential in a rat tibial bone defect model. **Methods**: AuNPs were synthesized and either left uncoated or coated with CS to enhance biological activity. Both formulations were examined for particle size, zeta potential, X-ray diffraction, and Fourier-transform infrared spectroscopy (FTIR). The resulting nanoparticles were integrated into gel bases, which were assessed for gel strength, swelling index, viscosity, and pH. The in vivo study involved surgically induced bone defects in the tibias of albino rats treated with either formulation. Healing outcomes were assessed via histological analysis, quantification of newly formed bone, immunohistochemical staining, radiographic imaging, and measurement of bone-related markers using RT-qPCR. **Results**: The CS-AuNP gel formulation demonstrated significantly improved bone regeneration compared to the uncoated counterpart, as evidenced by histological findings, increased bone volume in radiographs, stronger immunohistochemical expression of the VEGF angiogenic protein marker, and increased genetic expression of osteogenic markers. **Conclusions**: Incorporating CS-AuNPs into gel formulations offers a promising approach for enhancing bone healing. The superior performance of the CS-coated system highlights its potential as a promising localized therapy for managing bone defects.

## 1. Introduction

Untreated bone defects often lead to progressive resorption of the remaining bone tissue. The use of bone substitute materials to fill these voids can prevent further loss and provide a stable foundation for either immediate or delayed implant placement [1]. Structural deficiencies in the jawbone can develop due to various factors, including infection, tumor resection, or cyst removal. In the United States alone, approximately 50 million teeth are extracted each year, and numerous apicectomy procedures contribute to post-surgical bone loss [2]. Bone defects can also arise as a consequence of periodontal disease, which frequently results in tooth loss if left untreated. Defect filling is essential to preserve teeth or ensure sufficient bone for future prosthetic or implant rehabilitation [3]. Despite the clinical importance of maintaining bone volume, only a small proportion of patients receive regenerative or grafting interventions. Remarkably, nearly 95% of implant failures are attributed not to systemic health or financial constraints but to inadequate alveolar bone volume [4].

Over time, various strategies have been explored to manage bone loss in dental applications, though with differing degrees of success. To enhance outcomes, there is a strong need for agents that not only halt bone resorption but also actively promote new bone development [5]. The topical application of growth factors, such as bone morphogenetic protein-2 (BMP-2) and fibroblast growth factor (FGF), has shown potential in accelerating bone regeneration [6]. However, limitations, including local degradation and potential immune reactions, remain a challenge for these therapies [7]. An alternative approach involves the use of pharmacological agents that are known to influence bone metabolism, potentially offering a more accessible and safer solution. Bisphosphonates are a well-established class of drugs that inhibit bone breakdown by targeting the mevalonate pathway, which plays a role in osteoclast function [8]. Inhibition of this pathway disrupts osteoclast activity, thereby reducing bone resorption and promoting bone formation. However, bisphosphonates do not contribute to the formation of new bone tissue [9]. This highlights the ongoing need for treatments that provide a dual function of suppressing bone resorption while simultaneously encouraging new bone growth [10].

The integration of nanotechnology into modern healthcare has led to notable improvements in patient care and overall health outcomes. This has paved the way for the development of more advanced drugs, treatment methods, and diagnostic technologies. In dentistry, nanotechnology is especially important for developing high-performance dental tools and therapies [11]. Nanoparticles (NPs), which are smaller than 100 nanometers, offer precise control due to their tiny size and structural features. These particles interact more effectively at the molecular level compared to their larger counterparts. Their extensive surface area also allows for enhanced adaptability across various biomedical applications. For medical use, NPs must be non-toxic, non-carcinogenic, mechanically strong, and capable of withstanding the internal environment of the human body. Recently, an increasing number of studies have underscored the critical contributions of NPs to tissue engineering and regenerative therapies [12].

Gold nanoparticles (AuNPs) are widely utilized in medicine due to their versatility and tunable nanoscale properties. They play an important role in drug development, diagnostic procedures, and therapeutic approaches [13]. Recent research has highlighted their antimicrobial, anticancer, antioxidant, and anti-inflammatory properties, often with lower toxicity compared to other metallic nanoparticles. Their biocompatibility and simple synthesis make them suitable for a range of biomedical applications, including targeted drug delivery, medical imaging, and therapy [14].

In addition, AuNPs have demonstrated broad effectiveness in drug delivery systems, in enhancing antimicrobial and anticancer therapies [15,16,17], and in tissue engineering and regenerative medicine applications [11,12,18,19,20,21]. In bone regeneration, AuNPs promote osteoblast differentiation, improve scaffold properties, and exert immunomodulatory effects by reducing inflammation and accelerating tissue repair [21]. Despite these promising outcomes, limited data are available on their long-term biocompatibility and mechanical stability in dental applications, and the impact of surface modification on their clinical performance remains insufficiently explored. Therefore, further investigation of the use of AuNPs in bone defect models is warranted.

Chitosan (CS) is a widely used natural polymer for bone repair and tissue regeneration due to its osteointegrability, osteoinductivity, and ease of application. It has been applied in various forms, including scaffolds and gels, for bone tissue engineering. CS has been shown to promote bone regeneration by enhancing osteoblast differentiation, increasing collagen deposition and mineralization, and supporting angiogenesis for improved nutrient supply [22,23].

To overcome these limitations and improve regenerative performance, this study adopts an integrated design rather than a simple combination of components. In this system, chitosan functions both as a bioactive polymer supporting osteogenesis and antimicrobial activity and as a stabilizing matrix for AuNPs, enhancing their dispersion and biocompatibility. AuNPs contribute to cellular interactions and osteogenic stimulation, whereas the gel matrix ensures localized retention and sustained delivery at the defect site. This rational design enables synergistic interactions among the components, leading to enhanced overall bone regeneration efficacy.

Given the growing interest in nanotechnology-driven strategies for bone regeneration, this study aimed to develop novel AuNP- and CS-AuNP-based gel formulations for the localized treatment of bone defects. The current study is distinguished by the integration of AuNPs and CS, known for their biocompatibility and unique bioactive properties, into a gel system that enables sustained delivery directly at the injury site. To the best of our knowledge, the combined use of CS-AuNPs in a gel-based system for the treatment of bone defects has not yet been sufficiently studied. The proposed system is not merely a combination of individual components; it is designed to integrate the osteoconductive and antimicrobial properties of CS, the bioactivity and cellular interaction capabilities of AuNPs, and the localized delivery function of the gel matrix into a unified platform for bone regeneration. Chitosan coating was employed to improve the stability and biocompatibility of AuNPs, while the gel matrix was intended to enhance retention at the defect site and provide sustained local exposure to the nanoparticle system. This integrated design allows the advantages of each component to be amplified, resulting in improved stability, local bioavailability, and therapeutic efficacy. The formulation was extensively characterized for its physicochemical properties, and its therapeutic efficacy was evaluated using a surgically induced tibial bone defect model in rats. A comprehensive assessment, including histological analysis, quantification of newly formed bone, immunohistochemical staining, radiographic imaging, and assessment of bone-related markers, was conducted to determine the extent of new bone formation.

## 2. Materials and Methods

### 2.1. Materials

Gold (III) chloride trihydrate and low-molecular-weight CS (50,000–190,000 daltons) were purchased from Sigma-Aldrich, St. Louis, MO, USA. Sodium chloride and acetic acid were purchased from PioChem (Cairo, Egypt). Hydroxypropyl methylcellulose (HPMC) was purchased from Sigma Chemical Co. (St. Louis, MO, USA).

### 2.2. Methodology

#### 2.2.1. Preparation of Nanoparticles

AuNPs were prepared using a 100 mL sample of aqueous gold (III) chloride trihydrate (0.25 mM) containing NaCl in a 250 mL flask at room temperature. The solution was then boiled on a magnetic stirrer (DAIHAN Scientific, Wonju, Republic of Korea) at 400 rpm, and 2 mL of sodium citrate was added until a red color appeared. CS-AuNPs were prepared using a 0.2% (*w*/*v*) CS solution added to AuNPs at a ratio of 1:1 (*v*/*v*) [24,25].

#### 2.2.2. Characterization of Gold Nanoparticles by UV–Visible Spectroscopy

At wavelengths between 200 and 800 nm, the UV-vis spectra of the Au solution were measured using spectrophotometry (Jasco, UK model no. V-630, JASCO Corporation, Tokyo, Japan). A baseline measurement was obtained using quartz crystal cuvettes containing 2 milliliters of 1% sodium citrate to get the localized surface plasmon resonance (LSPR) spectra produced by AuNPs [26].

#### 2.2.3. Determination of Particle Size, Size Distribution, and Zeta Potential

The particle size, size distribution, and zeta potential were measured at 25 °C using a Malvern Zetasizer (Malvern Panalytical Limited, Malvern, UK). Freshly prepared dispersions of AuNPs and CS-AuNPs were analyzed after dilution (1:100) using distilled water, and the average size and zeta potential were determined [27].

#### 2.2.4. X-Ray Diffraction (XRD)

CS, AuNPs, and CS-AuNPs were subjected to X-ray diffraction (XRD) studies. For every crystalline and amorphous phase found in the material, distinctive X-ray patterns were acquired. In the 2θ range of 5° to 70°, nickel-filtered Cu kα1 radiation with a wavelength of 1.5106 Å and running at 35 KW and 20 mA was employed. Diffractograms of X-rays were acquired at a scanning rate of 1° (2θ)/min [28].

#### 2.2.5. Fourier Transform Infrared (FTIR) Spectroscopy

Fourier transform infrared spectra were recorded for CS, AuNPs, and CS-AuNPs to ascertain whether the AuNPs retained their characteristic peaks during encapsulation. Using a hydrostatic press, the examined samples were compacted into a disc along with dry potassium bromide powder. The scanning range was between 400 and 4000 cm^−1^ [28].

#### 2.2.6. Transmission Electron Microscopy (TEM)

The purpose of this procedure was to assess the morphology of the AuNPs and CS-AuNPs. AuNP and CS-AuNP solutions (100 μL) were applied to the coated copper grids and allowed to dry. The grids were air-dried before examination using TEM (JEOL JSM-6510 LV, Tokyo, Japan) [29].

#### 2.2.7. Gel Preparation

Hydroxypropyl methylcellulose (HPMC) at a concentration of 5% was used as the gelling agent. The polymer was gradually dispersed in distilled water at room temperature with continuous stirring using a magnetic stirrer (Thennolyne Corporation, Dubuque, IA, USA) until complete hydration was achieved. Subsequently, AuNPs were incorporated into the hydrated HPMC solution under constant stirring to ensure uniform distribution, resulting in a final AuNP concentration of 200 µg/mL (200 ppm) in both the G-AuNP and G-CS-AuNP gel formulations. The final volume was adjusted with distilled water, and the mixture was allowed to stand at room temperature until a homogeneous, translucent gel was formed [30].

#### 2.2.8. Measurement of the Gel Strengths of the Gold NP Gel

At 37 °C, the weighed quantities of G-AuNPs and G-CS-AuNPs were placed in a 100 mL graduated cylinder. Each gel had a 35 g weight on it. The time (in seconds) needed for the weight to drop 5 cm was used to determine the gel strength [30].

#### 2.2.9. Physical Appearance and pH

Visual inspection was performed on the produced gels to check for color, homogeneity, and phase separation [31].

The pH values of the prepared gels in distilled water were assessed using a digital pH meter. Measurements were performed in triplicate, and the averages were calculated [31].

#### 2.2.10. Swelling Index

The gel swelling rate was measured using a 1% agar gel plate. The original weight (W_0_) of each gel was recorded. The formulations were placed in a gel-surfaced Petri dish and maintained in an incubator at 37 ± 1 °C. The formulations were removed, cleaned with ordinary filter paper, and reweighed (W_t_) at one-hour intervals for up to six hours [32]. The swelling index was calculated using the following formula:

Swelling Index (SW)% = [(W_t_ − W_0_)/W_0_] × 100

where (SW) % = Equilibrium percent swelling;

W_t_ = Weight of swollen gel after time t;

W_0_ = Original weight of gel at zero time.

#### 2.2.11. Spreadability

To determine spreadability, 0.5 g of each gel formulation was placed inside a circle that was pre-marked with a diameter of 1 cm on a 20 × 20 cm glass plate, which was then placed on top of another glass plate. For five minutes, a 500 g weight was left to rest on the upper glass plate. It was observed that the gel spreading caused an increase in diameter [33].

#### 2.2.12. Viscosity

A rotating viscometer (cone and plate viscometer) was used to assess the rheological properties of gel formulations. Between the Roto Cone and the viscometer plate, one gram of each gel was applied. A temperature of 25 ± 0.5 °C was used to measure the viscosity of each sample. A constant change in the speed rate was applied to each sample. The rheological parameters, including viscosity, shear rate, and shear stress, were measured. Plotting the shear rate against shear stress allowed for the creation of a full rheogram. Three determinations were averaged to obtain the results [30].

#### 2.2.13. In Vitro Release Study

The in vitro release of AuNPs from the hydrogel matrix was evaluated using a dialysis membrane diffusion method. Accurately weighed hydrogel samples (equivalent to a known AuNP content) were placed in dialysis bags (molecular weight cut-off: 12–14 kDa), which were then immersed in 15 mL of phosphate-buffered saline (PBS, pH 7.4) at 37 ± 0.5 °C under continuous shaking at 60 rpm to simulate physiological conditions. At predetermined time intervals (1, 6, 24, 48, and 72 h), 1 mL of the release medium was withdrawn and replaced with an equal volume of fresh PBS to maintain sink conditions. The concentration of released AuNPs was quantified using UV–visible spectrophotometry at 526 nm. The cumulative percentage release was calculated based on the total initial nanoparticle loading [30].

### 2.3. Animals and Surgical Procedures: In Vivo Study

Thirty adult male albino rats, each weighing 150–200 g, were used in this study. The number of animals required was estimated based on previous studies [34,35]. The animals were housed in standard polypropylene cages and maintained under controlled environmental conditions, including a temperature of 22 ± 2 °C, relative humidity of 50–60%, and a 12 h light/dark cycle to mimic normal circadian rhythms. They were provided standard laboratory chow and water ad libitum throughout the study. All procedures related to animal selection, housing, feeding, and handling strictly adhered to the ARRIVE guidelines to guarantee ethical standards and animal welfare. The study was conducted after obtaining ethics committee approval from the Faculty of Pharmacy, The British University in Egypt (EX-2220). Rats were anesthetized using an intraperitoneal injection of Ketamine Hydrochloride (33 mg/kg body weight) and Xylazine Hydrochloride (13 mg/kg body weight). Following induction, a 1–1.5 cm linear incision was made below the tibial head to expose the bone on the lateral aspect of the right tibia. Under saline irrigation, a sterile round dental bur (size 6) was used at a low speed to create a monocortical bone defect that reached the bone marrow on the lateral surface of the right tibia. The defect was 4 mm in diameter and 2 mm deep [34].

The rats were allocated to five distinct groups (six rats/group). To minimize bias, rats were randomly allocated by a blinded veterinarian using an online randomization program that generated a random number sequence, ensuring an equal probability of assignment to each group. Additionally, cage positions were regularly rotated. In the Control group, the bone defect was left untreated and served as a negative control. The Gel group was treated with plain gel. In the other study groups, the bone defects were filled with G-CS, G-AuNPs, or G-CS-AuNPs. All materials were added locally to fill the bone defect during the surgical procedures, with an average standardized weight of the formulation between 6 and 7 mg/defect. Finally, the muscle layer was sutured with resorbable 4.0 catgut (Cirumédica, Cotia, SP, Brazil), and the skin was closed using interrupted 4.0 silk sutures (Ethicon/Johnson & Johnson, São José dos Campos, SP, Brazil).

After surgery, each rat received an intramuscular injection of ceftriaxone (20 mg/kg) and diclofenac sodium (75 mg/mL) once a day for three days. After one month, the tibiae were carefully dissected to remove the soft tissues. The specimens were then decalcified in a solution containing 12% EDTA (pH 7.2) for three weeks at 4 °C in phosphate-buffered solution (PBS) [35]. Rat codes were kept unidentified to the outcome assessors (histological and radiographic assessors).

#### 2.3.1. Histological Examination

Histological staining was carried out using haematoxylin and eosin (H&E) stain to visualize bone development. After decalcification, the specimens were carefully cleaned under running water. They were dried by exposure to increasing alcohol concentrations (50%, 60%, 80%, 90%, 96%, and absolute alcohol). The specimens were then embedded in paraffin wax blocks. Sections were cut from paraffin blocks using a microtome at a thickness of 3µ. The sections were transferred between distilled water and alcohol at concentrations of 96%, 70%, and 99%. The sections were stained with haematoxylin and eosin (H&E) for conventional histological analysis using a light microscope [36].

#### 2.3.2. Quantification of the Newly Formed Bone

The Masson Trichrome (MT) special stain revealed areas of new immature collagen synthesis with blue staining, indicating nascent bone development, whereas mature collagen appeared reddish. The images were loaded into an imaging software (Olympus Soft Imaging System Analysis, version 5.0). Morphometric analysis was utilized to measure the mean area% of blue-stained areas in five fields (×400 magnification) from each section. All sections were examined under a light microscope (BX60; Olympus, Tokyo, Japan) with a digital camera (Olympus E-330, Olympus Corporation, Tokyo, Japan) connected to a computer [37].

#### 2.3.3. Immunohistochemical Staining

Immunohistochemical staining was conducted using monoclonal mouse antibodies targeting VEGF, adhering to the manufacturer’s guidelines. For histomorphometric analysis, images were obtained from five fields at ×400 magnification for each histopathological section. Morphometric analysis involved determining the average area percentage of positively immunostained VEGF protein, as indicated by the brown color [38].

#### 2.3.4. Radiographic Examination

In this study, all rats were subjected to radiographic imaging immediately after surgery (baseline) by a blinded assessor and one month later using a digital X-ray machine located in the X-ray department of the Faculty of Dentistry at the British University in Egypt (BUE). The digital sensor of the X-ray system was placed under the tibia, and the tibia was imaged following the procedures and device settings described in a previous study [35]. Bone mineral density (BMD) was evaluated using ImageJ software (Version 1.53k, National Institutes of Health, Bethesda, MD, USA) [39].

#### 2.3.5. Bone-Markers Genetic Expression

Bone-related markers, including bone morphogenetic protein-2 (BMP-2) and runt-related transcription factor 2 (RUNX2), were measured in bone defect specimens using real-time quantitative polymerase chain reaction (RT-PCR) analysis. Bone tissue was processed using TRIzol reagent according to the manufacturer’s instructions. Briefly, bone specimens were ground in a mortar with Trizol reagent (Invitrogen, Thermo Fisher Scientific, Waltham, MA, USA) and left for 5 min to lyse the cells. Chloroform was added to the sample, and the aqueous layer containing the RNA was precipitated using propranolol. The resulting precipitate was resuspended in RNase-free water after washing with ethanol. The concentration and purity of the extracted RNA were measured by a NanoDrop spectrophotometer (NanoDrop, Thermo Fisher Scientific). cDNA was synthesized using the miScript Reverse Transcription Kit (QIAGEN, Hilden, Germany). cDNA synthesis conditions included 25 °C for 5 min, 42 °C for 60 min, and 70 °C for 15 min for inactivation. For cDNA amplification and quantification, real-time quantitative polymerase chain reaction (RT-qPCR) was performed using the miScript SYBR Green PCR Kit. Real-time quantitative polymerase chain reaction (RT-qPCR) was performed using the SYBR Green PCR Master Mix in a final volume of 20 μL per sample. The PCR thermal cycling parameters for mRNA were 2 min at 50 °C, 30 s at 95 °C, 35 cycles of 95 °C for 10 s (denaturation), 60 °C for 40 s (annealing), and 72 °C for 30 s (extension). A negative control containing all the components of the reaction except cDNA was included in each PCR run to detect contamination or non-specific amplification. The results were further normalized to the reference gene GAPDH and are displayed as fold-changes by applying the 2^−ΔΔCt^ technique. The experiment was repeated three times (*n* = 3). The primer sequences used for amplification were assessed for specificity using NCBI Primer-BLAST and are provided in Table 1.

#### 2.3.6. Statistical Analysis

Data were assessed for normality and homogeneity of variance using the Shapiro–Wilk test. Results are demonstrated as mean ± SD. One-way ANOVA was used to compare groups at the same time points, followed by Tukey’s test for pairwise comparisons. Statistical analyses were performed using GraphPad Prism version 7.00 (GraphPad Software, San Diego, CA, USA).

## 3. Results

### 3.1. Characterization of Gold Nanoparticles

The UV–visible spectrophotometer, a highly effective technique for detecting manufactured metallic nanoparticles, was used to characterize the generated AuNPs. Owing to the collective oscillations of the electrons at the surface of the nanoparticles that are connected with the electromagnetic field of the incoming light, AuNPs show a localized surface plasmon resonance band in the UV spectra at approximately 526 nm. The λmax of the CS-AuNPs was measured at 526 nm in this investigation; the fact that λmax remained constant suggests that CS did not affect the AuNPs (Figure 1).

### 3.2. Determination of Particle Size, Size Distribution, and Zeta Potential

The particle size values for AuNPs and CS-AuNPs were 33.21 ± 1.5 nm and 44.65 ± 2.3 nm, respectively. The size distribution values for AuNPs and CS-AuNPs were 0.549 ± 0.1 and 0.566 ± 0.09, respectively. The zeta potential values of AuNPs and CS-AuNPs were −30.1 ± 3.1 mV and 41.6 ± 1.2 mV, respectively.

### 3.3. X-Ray Diffraction (XRD)

The XRD patterns of CS, AuNPs, and CS-AuNPs were recorded and shown in Figure 2 for AuNPs, CS-AuNPs, and CS. The 2θ spectrum was recorded between 10° and 70°, and the AuNP results indicated the presence of three distinctly strong peaks at 38°, 45°, and 65°, while CS-AuNPs showed peaks at 31.8°, 38°, and 45.5°. CS showed a large amorphous peak extending from 20° to 30°, which is characteristic of its semi-crystalline nature.

### 3.4. Fourier Transform Infrared (FTIR) Spectroscopy

As shown in Figure 3, the FTIR spectra of pure CS revealed bands at 3355, 2865, 1589, 1375, 1062, 1024, and 565 cm^−1^, which allowed for the complete characterization of the starting material. The stretching vibrations of the O-H or N-H groups caused a broad band to appear in the spectra of pure CS between 3555 and 3150 cm^−1^. The stretching of the C-H groups is responsible for the peak at 2865 cm^−1^. The absorption peak at 1375 cm^−1^ is associated with the bending of N−H bonds (amide II) (NH2), whereas the band at 1589 cm^−1^ is associated with the vibrations of carbonyl bonds (C=O) (amide I) of O=C-NHR. CS’s of the glucosamine group in C-C stretching may be the cause of the noticeable absorption peak at 1062 cm^−1^. The C-O stretching is responsible for the absorption peak at 1024 cm^−1^. FTIR spectra of the AuNPs revealed peaks at 3265, 1637, 1393, 1278, and 606 cm^−1^. FTIR spectra of CS-AuNPs showed almost the same peaks as AuNPs, which are 3266, 1637, and 587 cm^−1^. FTIR spectra of AuNPs revealed peaks at 3266, 1637, 1393, and 587 cm^−1^. FTIR spectra of CS-AuNPs showed almost the same peaks as AuNPs, which are 3265, 1637, 1393, 1278, and 646 cm^−1^.

### 3.5. Transmission Electron Microscopy (TEM)

As illustrated in Figure 4, both AuNPs and CS-AuNPs exhibited a predominantly spherical morphology with a relatively uniform size distribution. The spherical morphology of the nanoparticles is favorable due to their high surface area and physicochemical properties. The TEM images show that the nanoparticles retained their structural integrity after coating with chitosan, as no alteration in particle shape was observed. The absence of morphological differences between the AuNPs and CS-AuNPs indicates that chitosan was deposited as a thin layer over the AuNP surface rather than forming aggregates. Additional TEM images obtained from different fields of view are provided in Supplementary Figure S1, further supporting the representative morphology and distribution of the nanoparticles.

### 3.6. Gel Strength, Spreadability, Physical Appearance, and pH

The mechanical and physical properties of the prepared gel formulations were evaluated to determine their suitability for application. According to the findings, the gel strengths of G-AuNPs and G-CS-AuNPs were 10.5 and 10.3 s, respectively. The similarity between these values indicates that the incorporation of chitosan-coated gold nanoparticles did not significantly alter the structural consistency of the gel matrix. Maintaining comparable gel strength is important because it reflects the ability of the formulation to retain its structure while still being easily applied.

Spreadability determines the ease with which the gel can be uniformly distributed. The spreadability results of the generated gel formulations were 7.67 ± 0.15 and 8.05 ± 0.18 for G-AuNPs and G-CS-AuNPs, respectively, indicating good spreadability for both formulations. This suggests that the gels possess appropriate rheological properties that allow easy application without excessive resistance. G-AuNPs and G-CS-AuNPs appeared as clear light pink, and their texture was smooth and non-greasy. The average pH of the G-AuNPs was 4.9 ± 0.1, while that of the G-CS-AuNPs was 4.7 ± 0.1.

A representative photograph of the chitosan-coated hydrogel is provided in the Supplementary Data (Supplementary Figure S2), as both plain and chitosan-coated hydrogels exhibited identical macroscopic appearance and handling properties.

### 3.7. Swelling Index

The swelling index is a key component of the bioadhesive properties of the gel. After swelling, the adhesion gradually weakened from its initial strength, which may last for up to six hours. The results are presented in Table 2.

### 3.8. Viscosity

Figure 5 shows the rheograms for G-AuNPs and G-CS-AuNPs, which illustrate that all developed gel formulations displayed non-Newtonian pseudoplastic flow with shear-thinning behavior, meaning that the system’s disarranged viscosity (the slope of the curve) reduced as the shear rate increased. When the shear stress increases, the molecules of the gelling material begin to align their long axes in the flow direction; this orientation reduces the internal resistance of the gelling material and, consequently, its viscosity.

### 3.9. In Vitro Release Profile of AuNPs-Loaded Hydrogel

The in vitro release profiles of AuNPs from the plain hydrogel (G-AuNPs) and the chitosan-coated hydrogel (G-CS-AuNPs) are presented in Supplementary Figure S3. Both formulations exhibited a time-dependent release pattern over 72 h. The plain hydrogel (G-AuNPs) showed a gradual increase in cumulative release, reaching approximately 15% at 1 h, 30% at 6 h, 40% at 24 h, 55% at 48 h, and 70% at 72 h. In contrast, the chitosan-coated hydrogel (G-CS-AuNPs) exhibited a slower release profile, with cumulative release values of approximately 10% at 1 h, 20% at 6 h, 30% at 24 h, 45% at 48 h, and 60% at 72 h. Overall, both formulations demonstrated controlled release behavior; however, G-CS-AuNPs showed a more sustained release pattern compared with G-AuNPs. This behavior may be attributed to the presence of the chitosan coating, which acts as an additional diffusion barrier and enhances nanoparticle retention within the gel matrix. The cumulative release profiles are presented in Supplementary Figure S3.

### 3.10. Histological Results

Microscopic examination of H&E-stained bone specimens after 4 weeks revealed variable amounts of new bone formation among the study groups (Figure 6A–E). The least amount of bone formation was detected in the Gel group, while the most mature histological picture of cancellous lamellar bone was detected in the G-CS-AuNPs combination group. The Control group showed newly formed immature woven bone. The islands of bone were widely spread with the invasion of mesenchymal elements. Osteocytes surrounded by Howship’s lacunae were observed in the bone matrix. Specimens of the Gel group showed sparse islands of immature woven bone with abundant connective tissue elements in between. A few Howship-like lacunae of osteocytes were also observed. In the G-CS group, bone specimens showed trabeculae of woven bone separated by bone marrow spaces filled with mesenchymal cells. Osteocytes residing in Howship’s lacunae were also detected in the bone matrix. G-AuNP specimens showed woven bone undergoing resorption with a mildly stained bone matrix, dilated osteocyte lacunae, and small bone marrow spaces. A part of the specimen showed trabecular lamellar bone with osteocyte lacunae. A reversal line (black arrows) was detected at the interface between the two bone surfaces. The combination group G-CS-AuNPs showed an almost normal architecture of cancellous lamellar bone with many osteocytes surrounded by Howship’s lacunae. The Haversian system was also detected.

All groups’ specimens revealed normal healing architecture with newly formed bone and only minimal inflammatory cell infiltration. No evidence of necrosis, abscess formation, foreign body giant cell reaction, or adverse tissue response was observed in the treatment groups, suggesting favorable local biocompatibility.

### 3.11. Morphometric Analysis of the Newly Formed Bone

Microscopic analysis using Masson’s trichrome staining revealed an increased presence of immature collagen, denoted by blue staining, in the G-AuNP and G-CS-AuNP groups compared to the G-CS alone and control groups (Figure 7A–E). Statistical analysis demonstrated that both G-CS and G-AuNPs significantly promoted the formation of new bone (immature collagen), with the G-CS-AuNP group exhibiting the highest significant increase, approximately a 1.7-fold increase, compared to the other study groups, as shown in Figure 7F. The mean area fractions of blue staining for the Control, Gel, G-CS, G-AuNPs, and G-CS-AuNPs were 9.02 ± 1.53, 8.96 ± 2.19, 19.79 ± 2.05, 44.42 ± 2.96, and 61.55 ± 4.81, respectively.

### 3.12. Bone Mineral Density Results

Radiographic images of the bone specimens showed that the control group possessed the lowest BMD, which appeared as a radiolucent area. The defect area became less radiolucent in the study groups, with the highest BMD in the combination group showing the least radiolucent defect area (Figure 8A–E). The mean percent change in BMD in the defect area among the study groups was calculated from time zero (day of surgery) to one month postoperatively. Consistent with our previous results, the G-CS and G-AuNP groups showed a significant increase in BMD%, with a higher percentage in the G-AuNP group. The G-CS-AuNP combination group showed the highest BMD% change compared to each material alone (*p* < 0.001) (Figure 8F). The mean BMD% change values of the Control, Gel, G-CS, G-AuNP, and G-CS-AuNP groups were 18.25 ± 3.62, 16.68 ± 3.73, 48.60 ± 6.89, 59.77 ± 4.67, and 81.43 ± 5.21, respectively.

### 3.13. Immunohistochemical Analysis of VEGF

Immunohistochemical analysis was performed to assess VEGF protein expression. Microscopic images revealed that the G-CS and G-AuNP groups possessed denser staining for VEGF compared to the control groups. Notably, the G-CS-AuNP group demonstrated more pronounced brown staining than either material alone (Figure 9A–E). Statistical analysis indicated that the mean fraction area of positively stained VEGF was highest in the G-CS-AuNP group, showing approximately a 1.5-fold increase compared to the AuNP group and a 2-fold increase compared to the CS group (Figure 9F). The mean fraction areas of positive immune staining in the Control, Gel, G-CS, G-AuNP, and G-CS-AuNP groups were 11.02 ± 0.78, 11.28 ± 1.15, 16.25 ± 1.10, 27.74 ± 2.28, and 40.97 ± 1.68, respectively.

### 3.14. Bone-Related Markers Genetic Expression

RT-PCR analysis indicated a significant increase in BMP-2 genetic expression in both the G-CS and G-AuNP groups compared to the control (*p* < 0.001) and Gel groups (*p* < 0.001), with the highest expression observed in the G-CS-AuNP combination group (*p* < 0.01) (Figure 10). The mean relative expressions of BMP-2 in the Control, Gel, G-CS, G-AuNP, and G-CS-AuNP groups were 1.039 ± 0.08, 1.13 ± 0.14, 1.86 ± 0.13, 2.48 ± 0.23, and 3.08 ± 0.14, respectively. Similarly, RUNX2 expression followed the same pattern; however, no significant difference was detected between the G-CS and G-AuNP groups (*p* = 0.079). The mean relative expressions of BMP-2 in the Control, Gel, G-CS, G-AuNP, and G-CS-AuNP groups were 1.12 ± 0.12, 1.07 ± 0.25, 1.74 ± 0.14, 2.23 ± 0.24, and 2.81 ± 0.22, respectively.

## 4. Discussion

Bone defects remain a major challenge in both the medical and dental fields, often resulting in significant bone resorption after surgical or therapeutic interventions. Although bone grafts are commonly employed to repair such defects, they present several limitations, including limited availability, donor site morbidity, and variable integration. Therefore, alternative bioactive materials capable of promoting bone regeneration are of growing interest [40]. In dentistry, the use of bone substitute materials has been shown not only to prevent resorption but also to maintain adequate bone volume for future restorative procedures [41]. The present study evaluated the regenerative potential of AuNPs and CS-AuNPs in a rat tibial defect model to determine whether their combination could enhance bone reconstruction.

Bone and dentin share key physicochemical characteristics, such as being mineralized connective tissues composed of hydroxyapatite crystals embedded within an organic, collagen-rich matrix. This similarity supports the use of tibial bone defects as an effective experimental model not only in orthopedics but also for studying bone defects in the oral cavity [42]. Dentin, a component of the tooth structure, exhibits structural and biological properties comparable to those of bone, including the presence of type I collagen and the ability to remodel [43]. Experimental studies have demonstrated that dentin grafts implanted into rat tibial defects undergo ankylosis and resorption and are subsequently replaced by newly formed bone, highlighting their osteoconductive and osteoinductive potential [44,45]. Moreover, dental pulp-derived stem cells have been shown to induce bone regeneration in ectopic and orthotopic bone models, including the rat tibia [46]. Therefore, although not a direct surrogate for dental tissues, the rat tibia provides a reliable and reproducible experimental model for evaluating biological reactions related to mineralized tissue repair and regeneration. The larger particle size observed in the CS-AuNPs was attributed to the chitosan coating, consistent with previous reports that CS increases the nanoparticle diameter [27]. The positive zeta potential confirmed the successful coating of AuNPs with CS, while XRD analysis verified the preservation of the crystalline gold structure. Minor peak shifts indicated uniform CS deposition on the AuNP surface. The additional feature observed at approximately 31.8° lies within the broad diffraction region of the chitosan matrix and may therefore be associated with the polymer background and structural interactions between chitosan and the incorporated gold nanoparticles rather than the crystalline gold phase. These findings align with earlier studies reporting similar structural characteristics [27,30,47]. The FTIR spectra of CS-AuNPs in the present study exhibited almost similar peaks to those of pure AuNPs, indicating uniform deposition of CS over AuNPs. The only differences between the FTIR spectra of CS-capped AuNPs and pure AuNPs are the slight shift in a broad band from 587 cm^−1^ to 646 cm^−1^ and the appearance of small peaks at 1393 and 1278 cm^−1^, which show that the amide group of chitosan interacts with the surface of AuNPs, resulting in successful coating [48]. The broad O–H/N–H stretching band shifted from 3355 to 3265 cm^−1^, while the amino group-related band shifted from 1589 to 1637 cm^−1^. Additional shifts were observed at 1375–1393 cm^−1^ and 565–646 cm^−1^. These spectral changes indicate alterations in the chemical environment of the hydroxyl and amino groups of chitosan resulting from their interaction with the gold nanoparticle surface. Moreover, the disappearance of the bands at 2855, 1062, and 1024 cm^−1^ and the appearance of a band at 1278 cm^−1^ indicate alterations in the chemical environment of the chitosan functional groups. Therefore, the FTIR results confirm the successful coating and stabilization of gold nanoparticles by chitosan. TEM images revealed a spherical morphology for both AuNPs and CS-AuNPs, indicating the homogeneous distribution of chitosan over the surface of AuNPs. This suggests that the chitosan coating process did not induce aggregation or structural deformation of the gold nanoparticles [49,50]. Both gel formulations, G-AuNPs and G-CS-AuNPs, appeared clear and non-greasy. The slightly higher spreadability observed in G-CS-AuNPs may be attributed to the interaction between chitosan and the gel matrix, which could slightly modify the internal structure of the formulation and enhance its flow characteristics. Such behavior is beneficial for topical drug delivery systems, as it facilitates the uniform distribution of active components across the surface [51]. The swelling index increased proportionally with HPMC hydration, with the highest swelling observed in the CS-containing formulation due to the combined hydrophilic properties of CS and HPMC [52,53]. Gel strength was investigated to assess the consistency and mechanical integrity of the hydrogel. The rheological profiles demonstrated pseudoplastic (shear-thinning) behavior, as evidenced by the decrease in viscosity with increasing shear rate. Pseudoplastic flow is a desirable characteristic for pharmaceutical hydrogel systems because it facilitates administration by reducing resistance to flow under applied shear stress. Following administration, the viscosity increases under low-shear conditions, which may enhance formulation retention at the target site, reduce displacement from the defect area, and support sustained local delivery of the incorporated nanoparticles. These characteristics are particularly advantageous for localized bone regeneration applications, where prolonged residence time may contribute to improved therapeutic performance. Furthermore, the viscosity of the gel formulations exhibited thixotropic behavior, with lower shear stress at any rate of shear on the down curve compared to the up curve. Thixotropy, also known as time-dependent flow, occurs when a gel requires a specific amount of time to restructure itself, which breaks down under constant shear conditions. This property is advantageous for topical or localized drug delivery systems, as it facilitates easy application and recovery of the gel viscosity post-administration [54,55].

The in vitro release study demonstrated sustained nanoparticle release from both hydrogel formulations throughout the experimental period. However, G-AuNPs exhibited a higher cumulative release than G-CS-AuNPs at all evaluated time points. The slower release observed for G-CS-AuNPs may be attributed to the presence of the chitosan coating, which acts as an additional diffusion barrier around the nanoparticles and increases interactions between the nanoparticles and the hydrogel matrix. These interactions may hinder nanoparticle mobility and reduce the diffusion rate through the swollen hydrogel network, resulting in a more sustained release profile. Both formulations exhibited an initial burst release, which is likely due to the rapid diffusion of nanoparticles located at or near the hydrogel surface. This was followed by a sustained release phase corresponding to the gradual diffusion of nanoparticles entrapped within the hydrogel matrix. The reduced release rate of G-CS-AuNPs suggests that chitosan coating enhances nanoparticle retention within the hydrogel and contributes to prolonged release behavior. From a therapeutic perspective, the prolonged release exhibited by G-CS-AuNPs may enhance local nanoparticle retention at the defect site and support sustained bioactivity during the bone healing process. The release rate observed in vitro may overestimate the actual release rate in vivo. The tibial defect environment provides a relatively static microenvironment with limited fluid turnover compared with the sink conditions and agitation employed during in vitro release testing. Consequently, diffusion of gold nanoparticles from the HPMC/chitosan matrix is expected to be slower in vivo, resulting in prolonged retention at the implantation site [56].

In our study, we induced standardized bone defects in albino rats with a diameter of 4 mm and a depth of 2 mm. Animal models remain the gold standard for evaluating newly developed materials and formulations for bone regeneration in the preclinical stage. This approach provides an opportunity to assess the efficacy of novel biomaterials in promoting new bone formation histologically, immunohistochemically, radiographically, and through bone markers’ genetic expression.

CS was chosen for our study due to its desirable properties, such as high osteoinductivity, osteointegrability, ease of application, antibacterial activity, and gradual biodegradability, making it an ideal candidate for bone tissue engineering applications. Several studies have demonstrated the positive influence of CS-based compounds on bone repair in animal models [57,58]. In the present study, microscopic examination after 4 weeks of H&E-and Masson trichrome-stained bone specimens revealed that both CS and AuNPs increased bone formation, with the greatest new bone formation in the combination group compared to each material alone and the control. These results coincide with those of Chevrier et al. [59], who studied the effect of CS on subchondral bone remodeling in drilled cartilage defects and revealed in their study that it increases vascularization and promotes granulation tissue formation. Their results, 2 weeks after surgery, showed a mixture of woven bone and fibrous tissue in CS-treated defects, and fibrous tissue was dominant in untreated defects. Our findings also align with those of Ezoddini-Ardakani al. [60], who demonstrated that CS stimulates bone formation, with woven bone detected in all CS-treated specimens, compared with limited new bone in the controls [60].

In the present study, both CS and AuNPs enhanced bone formation; however, the CS-AuNP combination showed the most pronounced induction of new bone, as confirmed by histological, immunohistochemical, and radiographic examinations. Our results were further confirmed by the increased genetic expression of bone-related markers, including BMP-2 and RUNX2, in the studied groups, with the same pattern. Several researchers have investigated the effects of CS on bone healing and have proposed hypotheses regarding its mode of action. For instance, Chernysheva et al. reported that CS stimulated bone reconstruction and accelerated bone regeneration induced by recombinant human bone morphogenetic protein-2 [61]. In another study, Budiraharjo et al. confirmed that coated and hydroxyapatite (HAP)-coated carboxymethyl chitosan (CMCS) scaffolds can act as osteogenic mediators for the induction of bone formation [62]. Mathews et al. suggested that CS can increase mineralization by upregulating associated genes as a mechanism of osteogenesis [63]. Li et al. proved that CS significantly induced osteogenesis at the graft–bone interface after histological examination [64].

In previous studies, nanoparticles, including AuNPs, have been investigated and proven to be alternative regulators of cell differentiation [65,66,67,68]. AuNPs have been reported to act as a new osteogenic agent that promotes bone regeneration [69]. In the current study, AuNPs alone induced woven bone formation with a mildly stained bone matrix, dilated osteocyte lacunae, and small marrow spaces, indicating early remodeling. Some regions showed newly formed lamellar bone with reversal lines at the interface between old and new surfaces. These results agree with previous findings showing that AuNPs can induce osteogenic differentiation of periodontal ligament stem cells (PDLSCs) in a size-dependent manner, with 45 nm AuNPs exhibiting superior effects compared to 13 nm particles [70]. These results also coincide with another study that confirmed that osteogenic differentiation of PDLSC sheets was enhanced by AuNP supplementation [71]. Previous in vitro studies suggested that nanoparticles accelerate osteogenic differentiation of bone marrow stem cells through the BMP-2/Smad, PI3K-Akt, and MAPK signaling pathways [72,73]. Other researchers have suggested that AuNPs increase the expression of ALP and deposition of calcium salts and upregulate the expression of osteoblast markers BMP-2, OCN, Col-1, and Runx-2 through the ERK/MAPK signaling pathway [74,75]. It was also reported that AuNPs downregulate the expression of osteoclast differentiation markers NFATC1, c-Fos, and TRAP, inhibit osteoclast formation by suppressing the RANKL-induced signaling pathway, and prevent OVX-induced bone loss [76].

Bone tissue engineering requires spatiotemporal interactions between bone tissue and the vascular network to preserve skeletal integrity. The integration of the scaffold with host tissues requires adequate vascularization to supply oxygen and nutrients to the regenerating bone. Vascularization forms a capillary network by differentiating progenitor cells into endothelial cells and vascular tissues [77]. A study by Bai et al. showed approximately a 14% increase in bone mineral density in a mouse model using AuNPs [78]. The study reported that mesenchymal stem cells differentiated into endothelial cells at the tissue repair site, creating a vascular network. In addition to cytokines, macrophages secreted growth factors such as BMP-2, TGF-β, and VEGF, which aided in bone formation and vascularization. Another recent study by Gao et al. combined AuNPs with other therapeutic agents to protect against bone loss and improve bone structure in osteoporotic rats [79]. On the other hand, a translational study previously reported that CS alone is sufficient to stimulate osteogenesis in femur bone defects in mice through upregulation of the RUNX2/OC/ALP signaling pathway [80].

In line with our study results regarding the CS-AuNP combination, Choi et al. reported that chitosan-conjugated AuNPs significantly enhanced calcium deposition and upregulated the expression of osteogenic marker genes in human adipose-derived mesenchymal stem cells without inducing cytotoxicity. These findings support the synergistic interaction between CS and AuNPs, reinforcing their potential as a localized bioactive delivery platform for efficient bone regeneration [66].

## 5. Conclusions

The findings of this study demonstrate the significant potential of CS-AuNPs as a localized bioactive therapeutic strategy for bone defect repair. The formulation exhibited favorable physicochemical characteristics and superior in vivo performance, with notable improvements in new bone formation, structural integrity, and biological response. While no in vitro cytotoxicity tests were performed, the in vivo findings did not show obvious adverse tissue reactions within the study period. To our knowledge, this is the first report to explore such a combination in the context of bone regeneration, introducing a novel delivery platform that integrates the bioactivity of AuNPs with the osteoconductive and biocompatible properties of CS. These outcomes pave the way for future translational investigations, positioning this hybrid system as a promising candidate for next-generation localized bone tissue engineering and regenerative therapy.

## 6. Limitations of the Study

A limitation of this study is the absence of detailed microstructural characterization of the hydrogel systems, such as scanning electron microscopy (SEM) or other advanced imaging techniques, to evaluate the internal porosity and network architecture. Future studies could be conducted to focus on incorporating ultrastructural characterization to better elucidate the internal gel morphology and its impact on performance.

To validate the effectiveness and comparability of the CS-AuNP formulation, it is crucial to compare its results with those of a material known for its bone repair capabilities. The analysis in this study is currently limited to a single time point; however, evaluating outcomes at multiple time points could provide evidence of the formulation’s accelerated effects. Conducting a safety analysis to identify any inflammatory response, along with serum biochemical and hematological evaluations, will be advantageous in confirming the safety of the material for future clinical applications.

## Figures and Tables

**Figure 1:**
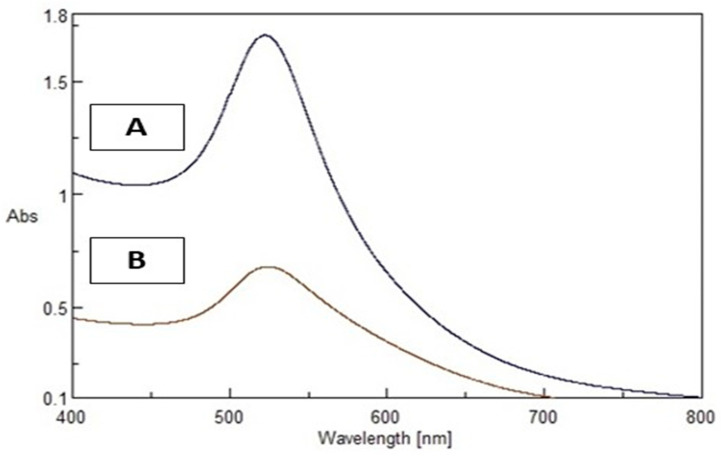
UV spectrophotometer analysis of (**A**) AuNPs and (**B**) CS-AuNPs.

**Figure 2:**
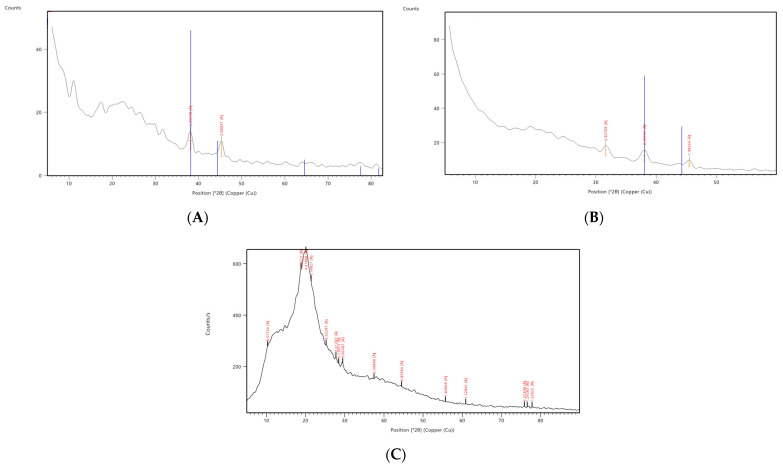
XRD patterns of (**A**) AuNPs, (**B**) CS-AuNPs, and (**C**) CS.

**Figure 3:**
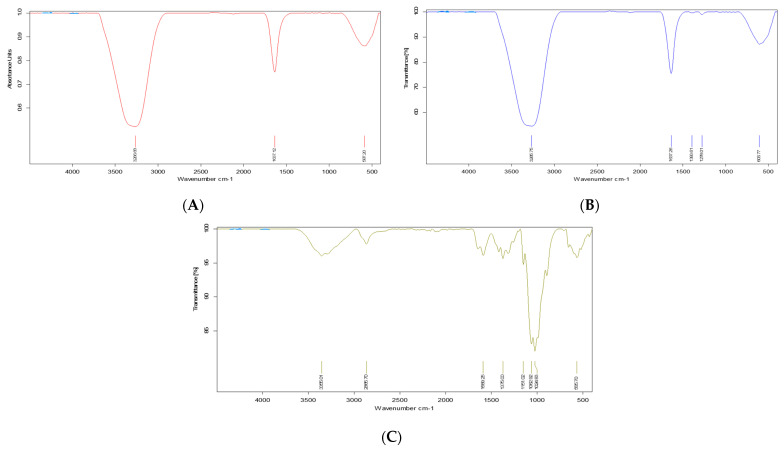
FTIR spectra: (**A**) AuNPs, (**B**) CS-AuNPs, (**C**) CS.

**Figure 4:**
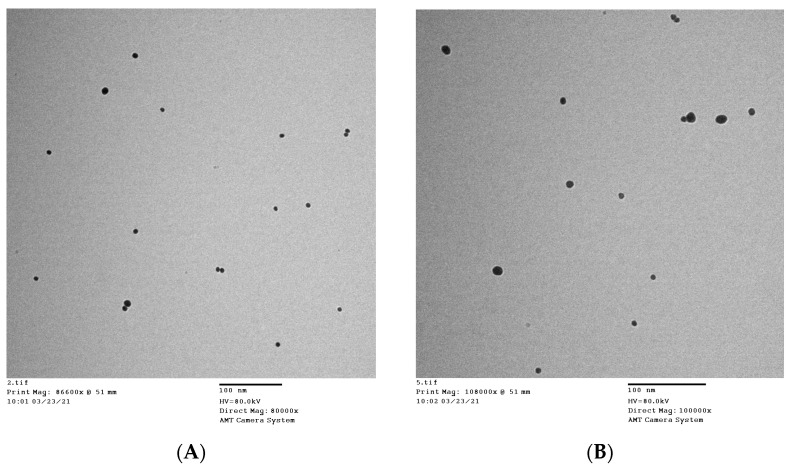
TEM images of (**A**) AuNPs and (**B**) CS-AuNPs.

**Figure 5:**
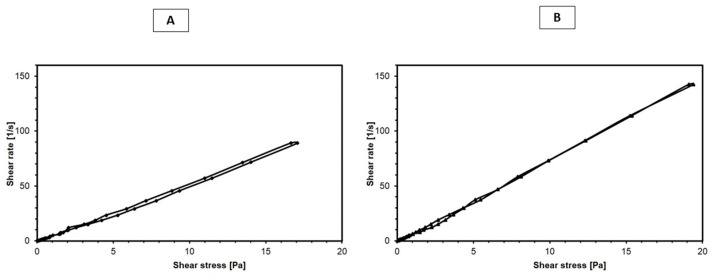
Rheograms of (**A**) G-AuNPs and (**B**) G-CS-AuNPs.

**Figure 6:**
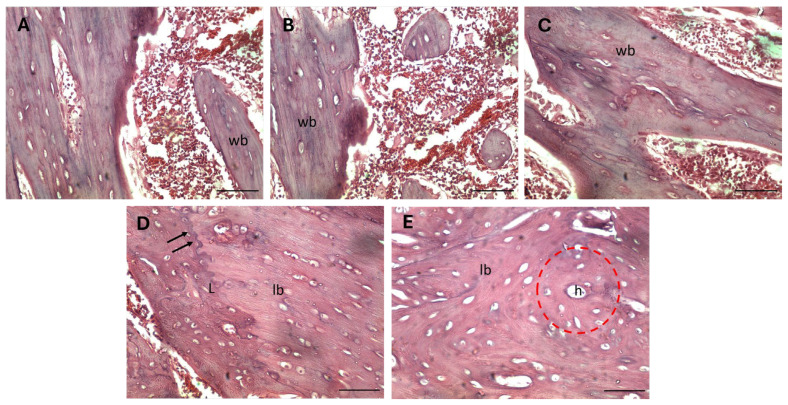
Histological patterns of rat tibial defects in the different study groups on day 30. Original magnification ×400, scale bar 500 µm. (**A**) Control group (negative control) showing invasion of connective tissue elements (CT) and formation of widely spread islands of woven bone (wb). Osteocytes (arrows) residing in their lacunae are seen in the bone matrix. (**B**) Gel group showing abundant mesenchymal elements (CT) surrounding sparse islands of immature woven bone (wb). A few osteocytes are observed in the bone matrix surrounded by their lacunae. (**C**) G-CS group showing trabeculae of woven bone (wb) separated by wide bone marrow spaces in between the trabeculae filled with mesenchymal cells (CT). (**D**) G-AuNP group showing the cancellous bone of tibia metaphysis transforming into lamellar bone (lb) with observed reversal lines (L). Small bone marrow spaces (CT) can be detected. Osteocytes (arrows) surrounded by their lacunae are seen in the bone matrix. (**E**) G-CS-AuNP group showing almost normal architecture of the lamellar bone (lb) of the tibia metaphysis with osteocytes residing in their lacunae. Haversian systems of mature lamellae are observed (h).

**Figure 7:**
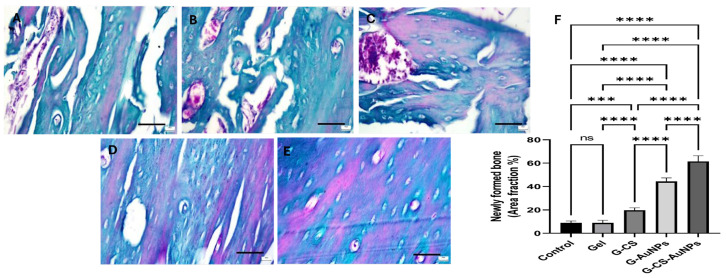
Microscopic images of bone specimens stained with Masson’s trichrome stain (original magnification ×400, scale bar 500 μm). The blue stain denotes the formation of new, immature collagen. (**A**) Control group (negative control) with the bone defect left empty (**B**) Gel group with the bone defect filled with plain gel (WB) (**C**) G-CS group with the bone defect filled with chitosan gel (**D**) G-AuNP group with the bone defect filled with nanogold gel (**E**) G-CS-AuNP group with the bone defect filled with the nanogold/chitosan combination (**F**) Statistical analysis of the newly formed bone among the study groups using one-way ANOVA test and Tukey’s post hoc analysis for intergroup comparisons. Results are demonstrated as mean ± SD, *n* = 6 rats per group. *** *p* < 0.001, **** *p* < 0.001, ns; non-significant. G, gel; CS, chitosan; AuNPs, nanogold.

**Figure 8:**
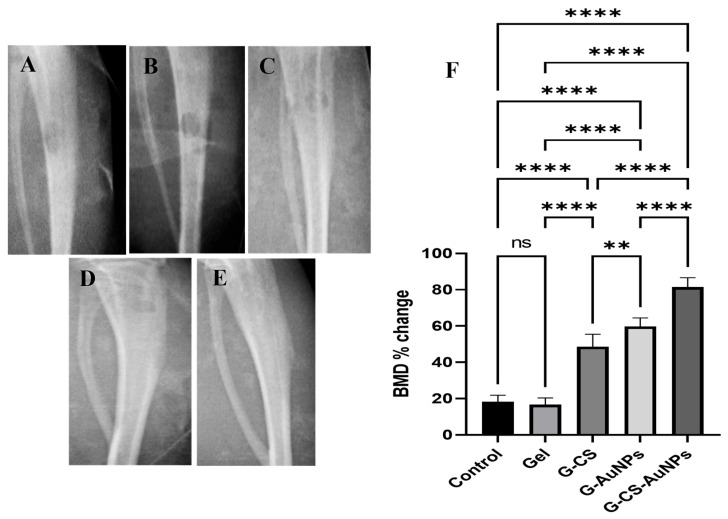
Radiographic images of the bone defects in the study groups one-month postoperatively (**A**) Control group (negative control) with the bone defect left empty (**B**) Gel group with the bone defect filled with plain gel (WB) (**C**) G-CS group with the bone defect filled with chitosan gel (**D**) G-AuNP group with the bone defect filled with nanogold gel (**E**) G-CS-AuNP group with the bone defect filled with the nanogold/chitosan combination (**F**) Statistical analysis of the change in BMD% among the study groups using one-way ANOVA and Tukey’s post hoc analysis for intergroup comparisons. Results are demonstrated as mean ± SD, *n*= 6 rats per group. *p* < 0.05 is considered statistically significant. ** *p* < 0.01, **** *p* < 0.001, ns; non-significant. G, gel; CS, chitosan; AuNPs, nanogold.

**Figure 9:**
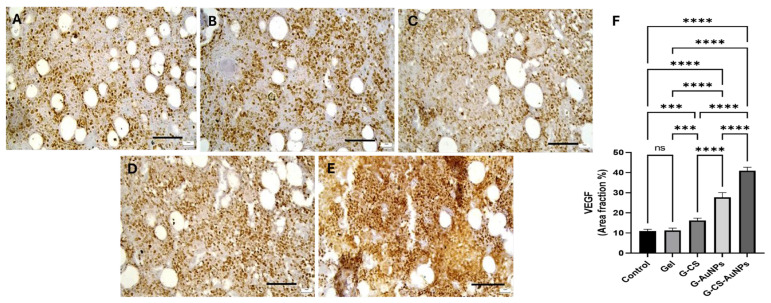
Images of positively stained VEGF (brown color) using immunohistochemical staining among the study groups (original magnification ×400, scale bar 500 μm). (**A**) Control group (negative control) with the bone defect left empty (**B**) Gel group with the bone defect filled with plain gel (WB) (**C**) G-CS group with the bone defect filled with chitosan gel (**D**) G-AuNP group with the bone defect filled with nanogold gel (**E**) G-CS-AuNP group with the bone defect filled with the nanogold/chitosan combination (**F**) Statistical analysis of the positively stained VEGF among the study groups using one-way ANOVA and Tukey’s post hoc analysis for intergroup comparison. Results are demonstrated as mean ± SD, *n* = 6 rats per group. *p* < 0.05 is considered statistically significant. *** *p* < 0.001, **** *p* < 0.001, ns; non-significant. G, gel; CS, chitosan; AuNPs, nanogold.

**Figure 10:**
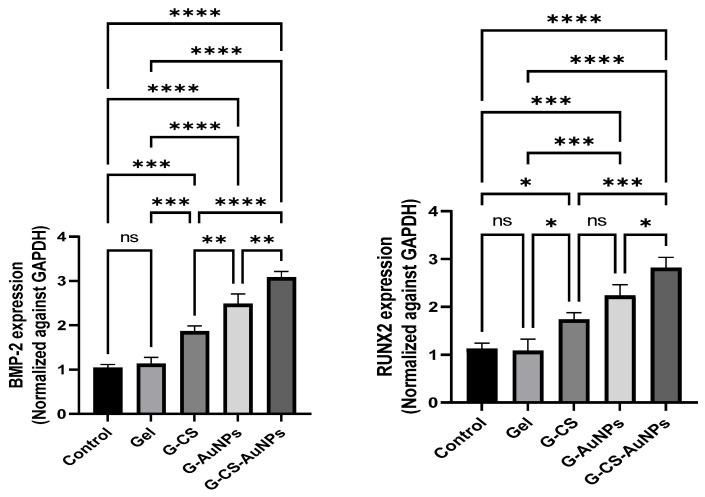
Bone-related markers’ genetic expression in the studied groups. Results are represented as mean ± SD (*n* = 6). Statistical analysis was performed using one-way ANOVA and Tukey’s post hoc analysis for intergroup comparisons. *p* < 0.05 is considered statistically significant. * *p* < 0.05, ** *p* < 0.01, *** *p* < 0.001, **** *p* < 0.001, ns; non-significant. G, gel; CS, chitosan; AuNPs, nanogold.

**Table 1:** Sequence of primers used in the study.

Marker	Forward Primer	Reverse Primer
BMP-2	TGTGGACTTCAGTGATGTG	TGGAGTTCAGGTGGTCAG
RUNX2	GACTGTGGTTACCGTCATGGC	ACTTGGTTTTTCATA ACAGCGGA
GAPDH	CTGGGCTACACTGAGCACC	AAGTGGTCGTTGAGGGCAATG

BMP-2: bone morphogenetic protein-2; RUNX2: runt-related transcription factor 2.

**Table 2:** Swelling Index values of G-AuNPs and G-CS-AuNPs.

Formulae	Time/h					
	1	2	3	4	5	6
G-AuNPs	24.45 ± 0.91	35.77 ± 0.89	44.71 ± 0.54	51.19 ± 0.64	59.82 ± 0.95	63.13 ± 0.69
G-CS-AuNPs	26.21 ± 1.11	38.42 ± 0.97	51.69 ± 0.83	57.18 ± 0.64	63.925 ± 0.51	69.34 ± 0.91

## Data Availability

The datasets generated or analyzed during the current study are available from the corresponding author upon reasonable request.

## Supplementary Materials

The following supporting information can be downloaded at: https://www.mdpi.com/article/10.3390/pharmaceutics18070843/s1. Supplementary Figure S1. Additional TEM micrographs of: (A) AuNPs and (B) CS-AuNPs. Supplementary Figure S2. Macroscopic appearance of the G-CS-AuNPs formulation after preparation. Supplementary Figure S3. In vitro cumulative release profile of G-AuNPs and G-CS-AuNPs over 72 h in PBS (pH 7.4) at 37 °C.
