# Supplementary material for: Dual-Functional Gel-Based Delivery of Chitosan-Coated Gold Nanoparticles for Accelerated Bone Healing in Defect Models
Source: Pharmaceutics. 2026 Jul 10;18(7):843. doi: 10.3390/pharmaceutics18070843 (PMC13414683; doi:10.3390/pharmaceutics18070843)
Supplement: Supplementary file 1 [file pharmaceutics-18-00843-s001.zip › pharmaceutics-4335936-supplementary.pdf]

Supplementary Data

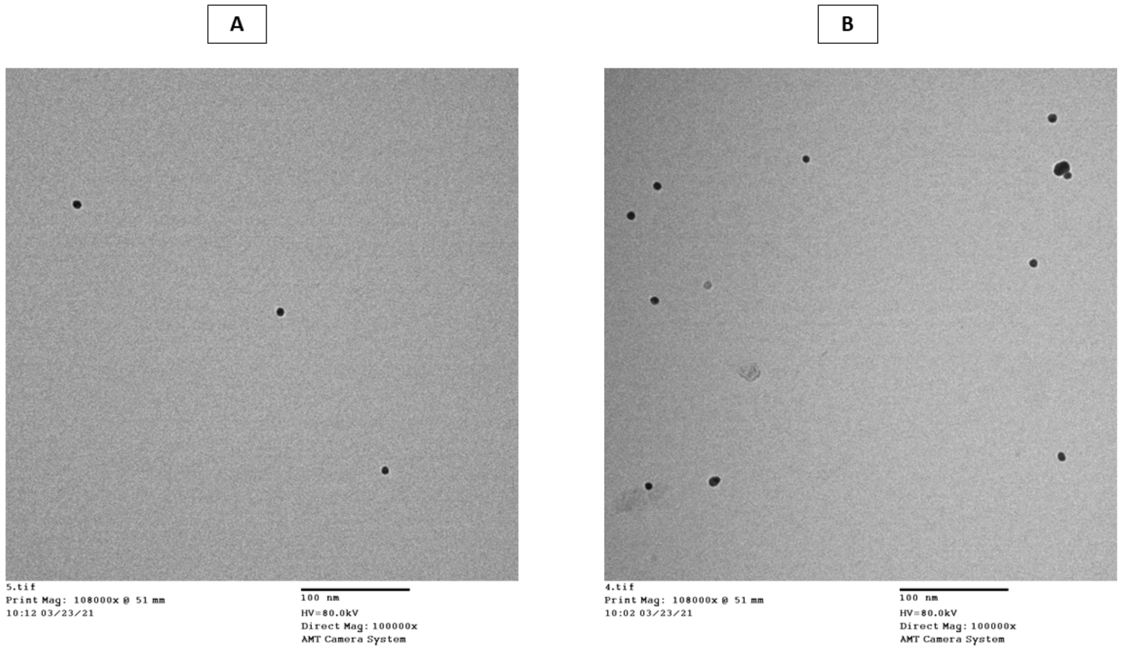

Supplementary Figure S1. Additional TEM micrographs of: A) AuNPs and B) CS-AuNPs

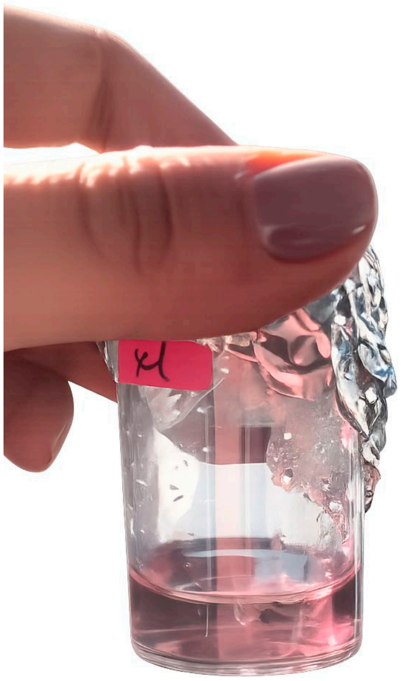

Supplementary Figure S2. Macroscopic appearance of the G-CS-AuNPs formulation after preparation.

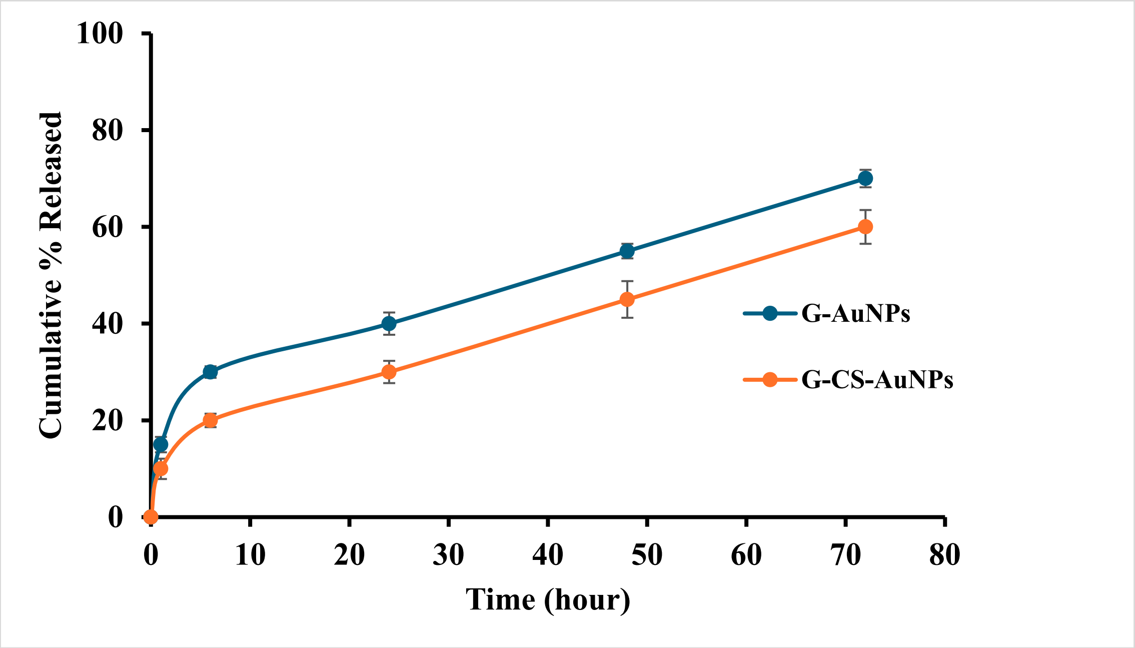

Supplementary Figure S3. In vitro cumulative release profile of G-AuNPs and G-CS-AuNPs over 72 hours in PBS (pH 7.4) at 37°C.
